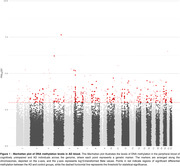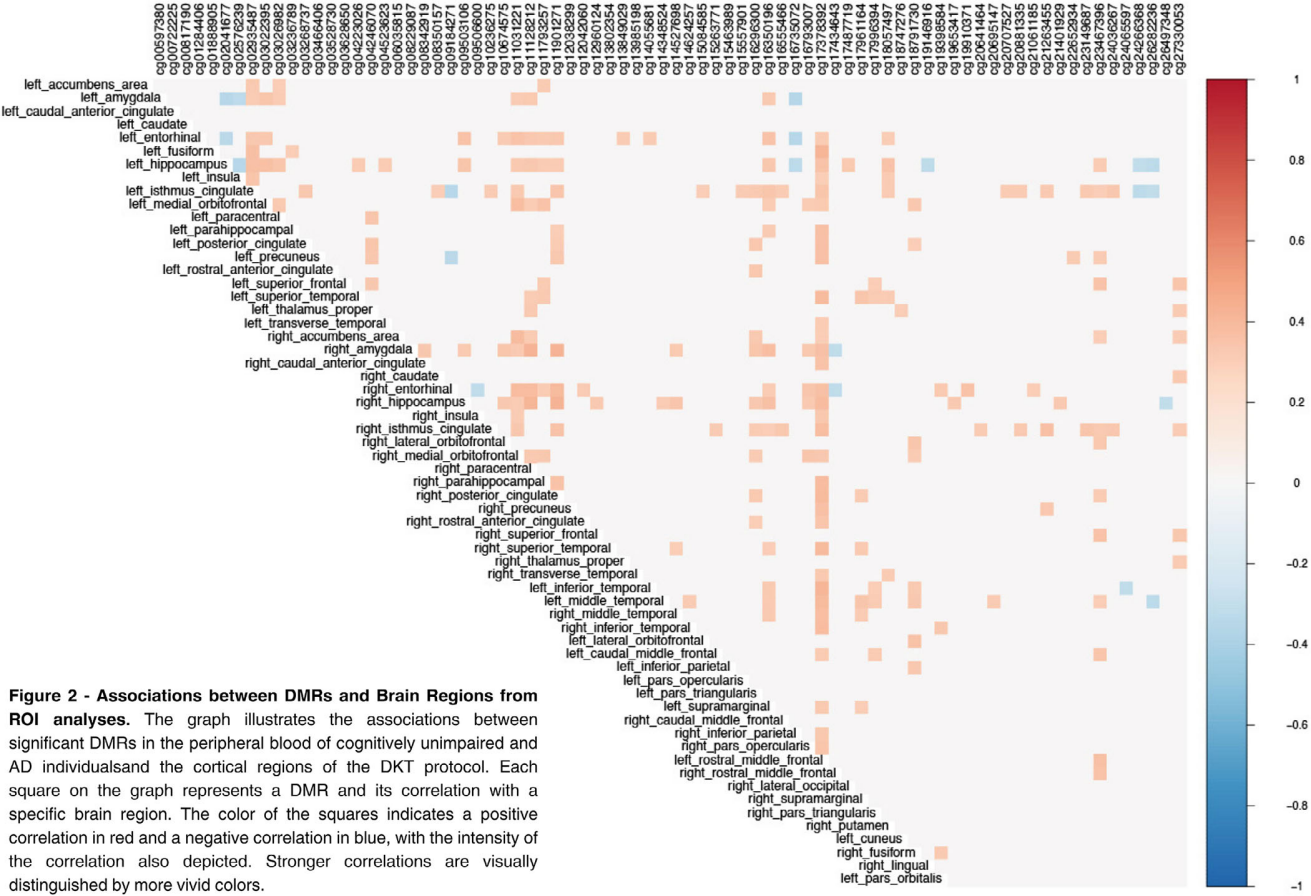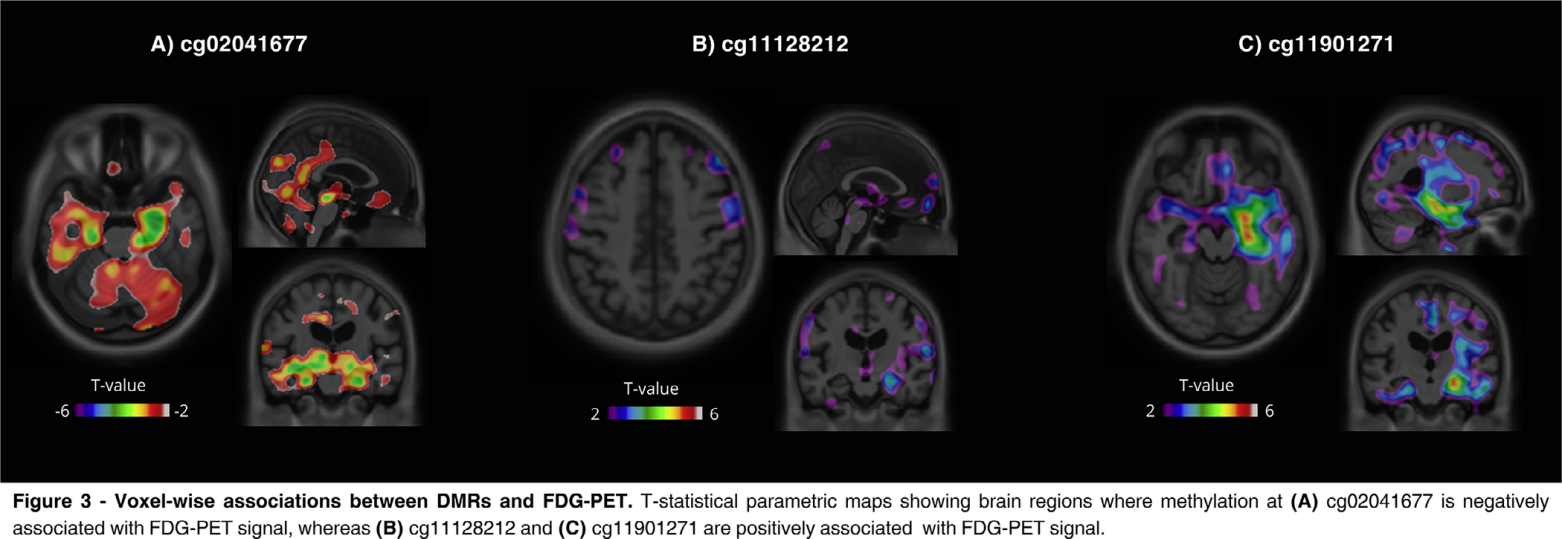# Associations between peripheral blood DNA methylation and FDG‐PET signal in AD individuals

**DOI:** 10.1002/alz.092675

**Published:** 2025-01-09

**Authors:** Lavinia Perquim, Marco Antônio de Bastiani, Luiza Santos Machado, Wyllians Vendramini Borelli, Guilherme Povala, Thomas Hugentobler Schlickmann, Tharick A. Pascoal, Pedro Rosa‐Neto, Alexandre Santos Cristino, Eduardo R. Zimmer

**Affiliations:** ^1^ Universidade Federal do Rio Grande do Sul, Porto Alegre, Rio Grande do Sul Brazil; ^2^ Memory Center, Hospital Moinhos de Vento, Porto Alegre, RS Brazil; ^3^ University of Pittsburgh, Pittsburgh, PA USA; ^4^ Translational Neuroimaging Laboratory, The McGill University Research Centre for Studies in Aging, Montréal, QC Canada; ^5^ Griffith Institute for Drug Discovery, Griffith University, Brisbane Australia; ^6^ Universidade Federal do Rio Grande do Sul, Porto Alegre Brazil; ^7^ Brain Institute of Rio Grande Do Sul, PUCRS, Porto Alegre, RS Brazil; ^8^ McGill Centre for Studies in Aging, Montreal, QC Canada

## Abstract

**Background:**

Epigenetics plays a crucial role in regulating genetic transcription and responding to environmental and lifestyle changes without altering the DNA sequence. Their dysregulation is associated with AD, presenting potential as blood biomarkers. However, no study has evaluated whether peripheral blood (PB) epigenetic biomarkers are associated with brain metabolism, indexed by FDG‐PET, a classic Imaging AD biomarker. Thus, we explore the associations between PB DNA methylation and FDG‐PET signal in the brain of cognitively unimpaired (CU) and AD individuals.

**Method:**

We evaluated CU=43 and AD=122 individuals from the ADNI cohort who underwent FDG‐PET imaging and PB DNA methylation analysis. Methylation data were analyzed using the minfi R package. Correlation analysis was performed with the statistically significant differentially methylated regions (DMRs) (p<0.005) and the regional FDG‐PET standardized uptake value ratio (SUVRs) values extracted with the DKT atlas. Voxel‐wise associations between FDG‐PET and DMRs were tested using linear regressions accounting for group, gender, age, and APOE4 status. The analysis was corrected for multiple comparisons using cluster‐wise RFT (p<0.05).

**Result:**

We identified 478 DMRs (Figure 1), multiple of them significantly associated with regional FDG‐PET SUVRs (Figure 2). The voxel‐based analysis demonstrated that DMR cg02041677, located in the ATE1 gene, was negatively associated with FDG‐PET signal in the left hippocampus, right orbitofrontal gyrus, and right medial temporal gyrus (tmax=‐4.28, ‐4.04, ‐3.58, respectively; p‐value<0.001). The cg11128212, in the intron, nearby two lncRNA (ENSG00000289046, ENSG00000274591), was positively associated with brain metabolism in the left hippocampus, left temporal pole, and left middle temporal gyrus (tmax=4.08, 3.98, 3.80, respectively; p‐value<0.001) while the cg11901271, located in intron, nearby of a LncRNA (ENSG00000287358), showed positive correlations with FDG‐PET in the Left hippocampus (tmax=5.35, respectively; p‐value<0.001) (Figure 3).

**Conclusion:**

Here, we show that PB DMRs exhibited a significant pattern of association with brain glucose metabolism in vulnerable AD regions. LncRNAs are important transcriptional regulators, the methylation could impact gene expression in AD and present potential as blood biomarkers.